# Perceptual-vision training as a strategy for healthy aging in adults with intellectual disability: a study protocol

**DOI:** 10.3389/fpsyg.2025.1526826

**Published:** 2025-04-24

**Authors:** Luca Cavaggioni, Damiano Formenti, Paolo Castiglioni, Luca Paolo Ardigò, Giampiero Merati

**Affiliations:** ^1^Department of Biotechnology and Life Sciences, University of Insubria, Varese, Italy; ^2^IRCCS Fondazione don Carlo Gnocchi, Milan, Italy; ^3^Department of Teacher Education, NLA University College, Oslo, Norway

**Keywords:** down syndrome, cognitive performance, intellectual impairment, visual training, physical activity

## Abstract

Aging leads to changes in motor-cognitive performance. Despite the importance of physical activity for healthy aging and the need for health promotion interventions in individuals with intellectual disability (ID), there is a lack of empirically strategies for promoting health in adults with ID. Therefore, we are conducting a clinical trial (NCT06628999 on clinicaltrials.gov) to investigate a strategy for promoting healthy aging by targeting physical and cognitive domains in ID individuals. The present work aims to provide a detailed account of the study protocol of the proposed trial to promote transparency and dissemination of the ongoing research. Specifically, this project will study the effect of a vision training program on cognitive performance and physical fitness in adults with ID. A secondary aim is to examine the association between mental and physical performance outcomes. Participants will be randomly allocated into a vision training group (VT, *n* = 28), a vision training-detraining group (VTD, *n* = 28), and a control group (C, *n* = 28). During the first 8 weeks, the VT and VTD groups will follow the same training protocol (based on vision oculomotor exercises combined with postural control exercises). From weeks 9 to 16, the VT group will continue the training protocol, whereas the VTD group will abstain from training (detraining). Cognitive performance and physical fitness will be assessed at baseline, mid- and post-training intervention. Overall, a vision training program can positively impact various aspects of life for individuals with ID promoting autonomy, and social integration to counteract the aging process.

## 1 Introduction

The United Nations expects that by 2050, a fifth of the global population will be older, with 15% of the population having some form of disability (Santos et al., 2022). Healthy aging involves developing functional abilities for wellbeing in older age and encompasses physical capabilities, cognitive functioning, metabolic health and psychological wellbeing (John et al., 2023). Aging is linked to changes in the locomotor system and reduced cognitive processing, leading to deterioration in motor-cognitive performance. In this sense, research has consistently demonstrated the crucial role of physical activity in improving physical fitness and preserving cognition in healthy older individuals (Erickson et al., 2019). However, as the majority of the literature focusedon healthy aging among the general population (Wollesen et al., 2020), there is also a need to focus on wellness promotion among individuals with intellectual disabilities. While most adults with cognitive disabilities have relatively few life co-existing conditions, some of them may experience severe health issues that impact their wellbeing (Reynolds et al., 2019). Compelling evidence suggests that adults with intellectual disabilities generally face unhealthy lifestyle habits, inadequate physical activity or poor nutrition health outcomes compared to their able-bodied individuals (McGuire et al., 2007). To sum up, a better comprehension on which type of intervention could contribute to ameliorate healthier old age outcomes for adults with cognitive impairments is fundamental.

Intellectual disability (ID) represents a heterogeneous condition based on the cognitive deficit and a wide range of comorbidities with a negative impact on aging (Krahn and Fox, 2014). ID includes some genetic syndromes (e.g., Down syndrome and Fragile X syndrome) with a remarkable effect on intellectual and adaptive behavior during childhood (U.S. National Library of Medicine, 2024), leading to deficits in motor development (Belluscio et al., 2019), sensorial context (Chokron et al., 2020) and visuospatial cognition (Giuliani et al., 2011; Wilkinson and Light, 2014). Notably, vision and eye tracking play a key role in spatial orientation, stimulating several brain areas (Henderson, 2003) and people with ID display anomalous visual movements, spatial organization and cognitive performance (Giuliani et al., 2011). In addition, an earlier acquisition of essential visual functions may have a protective effect on subsequent intellectual development (Dale and Sonksen, 2002).

With this in mind, multicomponent motor-cognitive training is a promising exercise category that stimulates multiple physical and cognitive skills, showing more remarkable similarities to daily life activities. In this sense, vision training with oculomotor eye-tracking stimuli has positively affected cognitive functions (Edlin and Lyle, 2013; Formenti et al., 2019). In older people, gross–motor coordination training and cognitive tasks counter age-related cognitive decline (Forte et al., 2023). Conversely, motor-cognitive interventions can also positively affect global cognition and inhibition (Wollesen et al., 2020). A recent review analyzed current interventions to support healthy aging in individuals with ID, highlighting physical activity as a crucial factor for healthy aging while emphasizing the need for more practical health promotion strategies to enhance the wellbeing of individuals with ID (Santos et al., 2022). However, there is a paucity of literature addressing feasible strategies that target both the physical and cognitive domains in individuals with ID to counteract the age-related decline. Therefore, we are conducting an interventional randomized clinical trial to investigate a potential strategy for promoting healthy aging by targeting physical and cognitive outcomes in individuals with ID. Aim of the present work is to provide a detailed account of the study protocol of our clinical trial to promote transparency and dissemination of the ongoing research. Specifically, the proposed protocol aims to investigate the effects of a 16-week vision training programme on cognitive and motor performance in individuals with ID.

### 1.1 Study objectives

The overall main purpose of this research project is to determine the effectiveness of perceptual-vision training on cognition and physical functioning in individuals with intellectual disabilities.

Specifically, the primary endpoint is to identify the role of eye training on cognitive performance in terms of inhibitory control. The secondary endpoints are to verify the contribution of vision training on postural control, upper and lower body strength and agility outcomes. The tertiary endpoint aims to verify any potential effect of age, reinforcing the concept of promoting healthy aging.

Our primary hypothesis is that perceptual vision training intervention will promote a greater increase in cognitive and motor performance compared to the usual care control group. In addition, the secondary hypothesis is that a period of detraining could slightly attenuate the training-induced gains achieved during the previous intervention period. The third hypothesis is to detect whether there is an age-related effect on the outcomes measured.

## 2 Material and methods

This study was approved by the University of Insubria Ethics Committee (protocol number 0119168, date 12.03.2024) and registered in ClinicalTrials.gov (NCT06628999) in compliance with the Helsinki Declaration. Written informed consent will be obtained from patients following the tenets of the Declaration of Helsinki on studies with human participants (Holt, 2014).

### 2.1 Study population

Participants will be recruited from local communities dwelling in Northern Italy. The inclusion criteria include age between 40 and 70 years with a low-to-mild intellectual disability (Intellectual Quotient, IQ = 51–69) defined with the Wechsler Scale of Intelligence (WAIS-IV), a scale particularly suited for individuals with an age range from 16 to 90 years composed by four sub-tests that would provide a comprehensive general intelligence index. The exclusion criteria are (a) inability to understand essential verbal communication, (b) strict dependence on personnel or assistive support devices, (c) presence of concomitant sensorial or physical impairments and (d) presence of behavioral problems or any other clinical condition that may compromise the practice of regular physical activity.

### 2.2 Study design

**Figure 1:**
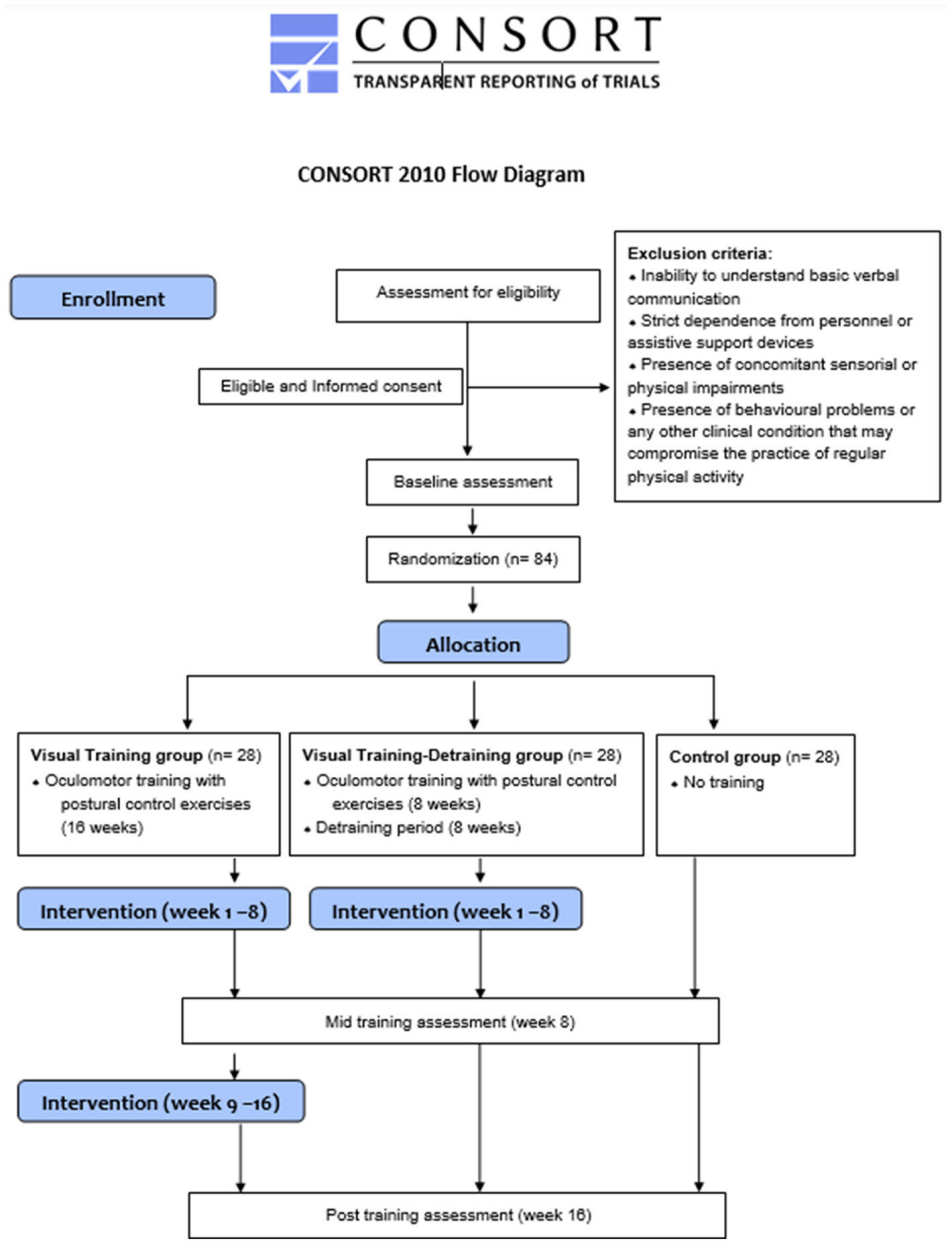
CONSORT diagram of the research.

This project is a three-arm, parallel, randomized controlled trial. In detail, Figure 1 highlights the flow diagram of the overall design setting. At the beginning, participants will have their body mass and height measured. After that, a 1-week familiarization period will be implemented during which participants will adapt to the exercise techniques used throughout the study. Following 1 week of baseline testing, participants will be randomly assigned to three groups: (i) a Visual Training Group (VT, *n* = 28) that will perform oculomotor exercises combined with postural control exercises (Formenti et al., 2019) for 16 weeks; (ii) a Visual Training-Detraining group (VTD, *n* = 28) that will follow the same training protocol of VT for the first 8 weeks, abstaining from any training for the following 8-weeks period (from week 9 to week 16) and (iii) a control group (C, *n* = 28) in which no intervention will be implemented. Randomization will be achieved using a random-number generator (https://www.random.org using a 1:1:1 ratio) and to ensure adequate allocation concealment this procedure will be carried out by a blinded external researcher. An independent researcher will conduct the allocation sequence until the interventions are assigned. A research team collaborator will enroll and assign participants to the three groups. Baseline testing will be repeated after 8 weeks (mid-training testing) and at the end of week 16 (post-training testing).

### 2.3 Intervention protocol

**Figure 2:**
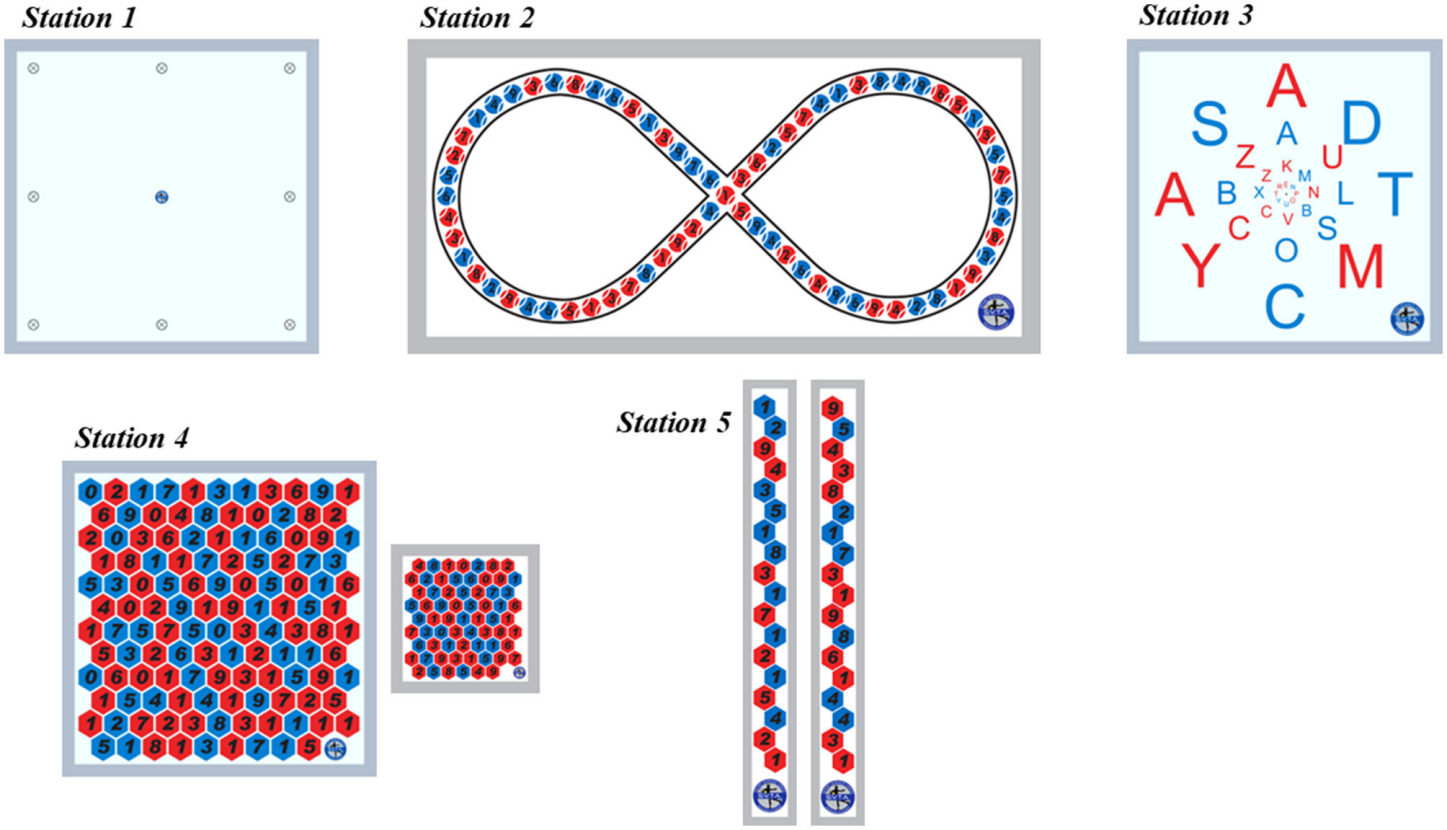
Vision training panel kit (reprinted with permission by Formenti et al., 2019).

The intervention programs for VT and VTD groups will be performed using the premium equipment of the S.V.T.A. training kit (S.V.T.A. method©, Carmagnola, Italy). This method combines visual skills (e.g., peripheral vision, saccadic eye movements, accommodation and vergence skills) with postural control actions. Each training session will be structured in a circuit-training format composed of 5 stations in which participants will perform the prescribed exercises for 6 min. Each station will be characterized by different panels fixed on a wall about 1.5 m from ground level, with colors, letters, and numbers as targets. VT and VTD groups will perform the same motor-cognitive oculomotor training combined with postural control exercises structured in four incremental sub-phases, as proposed by Formenti et al. (2019): phase 1 (weeks 1–4), phase 2 (weeks 5–8), phase 3 (weeks 9–12) and phase 4 (weeks 13–16). The difficulty of the oculomotor task increases progressively from Phase 1 to Phase 4 (for example, from bipedalism to monopodial stance and from close to far). The training circuit is composed of 5 stations (represented in Figure 2):

*Station 1*. A square board with nine points on a wall. Participants must move their gaze on external points in clockwise and anticlockwise directions, maintaining their focus on the central point.

*Station 2*. A rectangular board with an infinity profile on a wall. The infinity profile comprises a sequence of randomized red and blue points. Participants have to move their gaze from one point to another following the infinity profile while pronouncing the color of the point identified.

*Station 3*. A square board of four circles formed by a random sequence of red and blue letters fixed on a wall. Participants must move their gaze from one letter to another while pronouncing the identified one, maintaining the visual focus on the central point.

*Station 4*. A square board of 81 cells is fixed on the wall. Red or blue cells contain a random number from 0 to 9. Each participant holds a board comprising an image like the one on the wall. Participants have to read the numerical digit on the board in their hands and localize the same numerical digit on the board on the wall.

*Station 5*. Two boards with black-colored numerical digits (0 to 9) are fixed vertically on the wall. Participants must read digits while shifting their gaze from one board to another in mediolateral and sagittal directions.

### 2.4 Measurements and procedures

#### 2.4.1 Anthropometric variables

Body height and mass will be measured to the nearest 0.1 cm and 0.1 kg, respectively, using a beam scale (SECA 220, Hamburg, Germany).

#### 2.4.2 Cognitive performance

**Figure 3:**
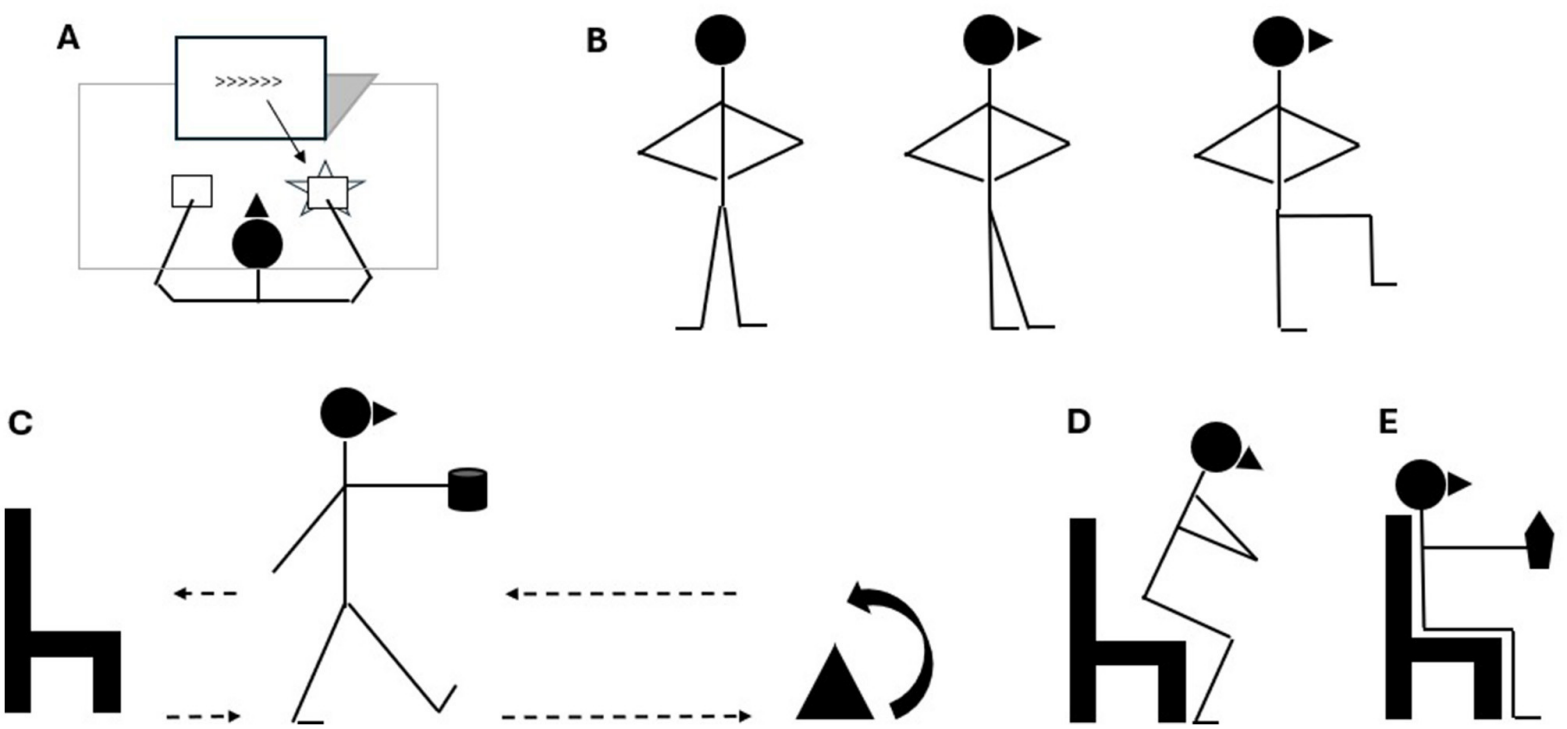
Testing procedures pictorial setting. **(A)** Cognitive performance, Flanker Task test. **(B)** Postural control, Modified Balance Error Scoring System. **(C)** Agility, Time Up and Go test. **(D)** Lower-body muscle strength, Five Repetition Sit-to-Stand test. **(E)** Upper-body muscle strength Handgrip Strength Test.

Inhibitory control will be measured by the Flanker Task, previously used in ID individuals (Van Biesen et al., 2017) (Figure 3A). Participants will be requested to indicate the direction of a left- or right-pointing target arrow as quickly as possible while ignoring two flanking arrows on each side pointing in the same or opposite direction. The task includes two conditions: congruent and incongruent. The congruent condition consists of trials in which both the target arrow and the four flanking arrows are pointed in the same direction (left: <<**<**<< or right: >>**>**>>). The incongruent condition consisted of trials in which the flanking arrows point in the opposite direction of the target arrow (<<**>**<< or >>**<**>>). Participants have to press button A on the keyboard when the target arrow points to the left (i.e., <) and L when the target arrow points to the right (i.e., >). Two sets of 50 trials each will be randomly proposed. The mean response time of the correct responses and the percentage of correct responses will be computed for each condition as the outcome.

#### 2.4.3 Physical fitness

Different biomotor abilities will be measured and visually shown in multipaneled Figure 3. Postural control will be screened by the modified Balance Error Scoring System (M-BESS) performed in an upright stance while conserving stability in three positions for 20 s (i.e., bipedalism, tandem or monopodial) on a firm surface (Figure 3B). The examiner will count the person's errors while maintaining the proper stance (Azad et al., 2016).

The integration between the agility domain and cognitive performance will be assessed by the time up-and-go test (TUG) (Figure 3C). Outcomes of the TUG test are: (i) the time a participant takes to get up from a chair, to walk and turn around a cone positioned 3 m far away and to walk backwards to the chair again while carrying a full glass of water in one hand (Maranhão-Filho et al., 2011) and (ii) the number of times the participant spills water on the ground (Kachouri et al., 2024).

Lower-body muscle strength will be assessed using the five-repetition sit-to-stand (5STS) by asking the patient to rise and sit down for five consecutive repetitions with arms crossed over the chest. This procedure is particularly suited to patients with disabilities (Polidori et al., 2024). Performance time in seconds will be obtained using a stopwatch (Figure 3D).

Upper-body strength will be detected with the handgrip strength test (HST), with the patient sitting on a chair while exerting the maximum isometric grip (Mathiowetz et al., 1985) (Figure 3E). A Camry dynamometer (CAMRY EH101, Sensun Weighing Apparatus Group Ltd, Guangdong, China), which provided excellent validity and reliability among adult-aged community-dwelling individuals (Huang et al., 2022), will be used to detect the maximal handgrip strength.

It is worth noticing that for M-BESS, TUG, 5STS, and HST procedures, the same tester will conduct three trials while respecting an interval rest of 60 s to detect the consistency and reliability of the measures.

### 2.5 Statistical analysis

The sample-size calculation is established based on a previous study (Formenti et al., 2019) on the primary outcome (incongruent condition of the Flanker task) using G^*^Power software. From a priori power analysis, observing a sample size of up to 84 individuals (β = 0.9) is requested, respecting a large effect size (Cohen's f equal to 0.4) with a type I error rate of α = 0.05. All data will be presented as mean ± standard deviation. The dataset will be tested for normal distribution using Shapiro–Wilk's normality test. Based on the study's design, the primary independent endpoints are the treatment groups and time. A two-way (treatment × time) repeated measures ANOVA will be used on each outcome variable to determine the interaction between the three time points (baseline, 8 weeks, 16 weeks) and groups (VT, VTD, C). If an interaction exists, the simple effect and pairwise comparisons will detect differences between groups at each time point. Subsequently an additional age stratification and comparison will be performed to check any age-related difference. If a main effect is detected, data will be analyzed using a *post-hoc* analysis (Bonferroni test). The significance level will be set at *p* < 0.05. Partial eta squared (Part 2) effect size will be used to estimate the magnitude of the difference within each group, respecting a threshold for small (0.01), moderate (0.06) and large effects (0.14) (Cohen, 2013).

If the Shapiro-Wilk's normality test is not passed, non-parametric tests will be applied. The Friedman Test will be performed to detect differences between groups over time in both primary and secondary outcomes. In addition, to examine where the differences occur, a Wilcoxon Signed Rank test with Bonferroni adjustment will be used as a *post-hoc* analysis. Also, in these tests, the significance level will be set at *p* < 0.05.

## 3 Expected results and discussion

**Figure 4:**
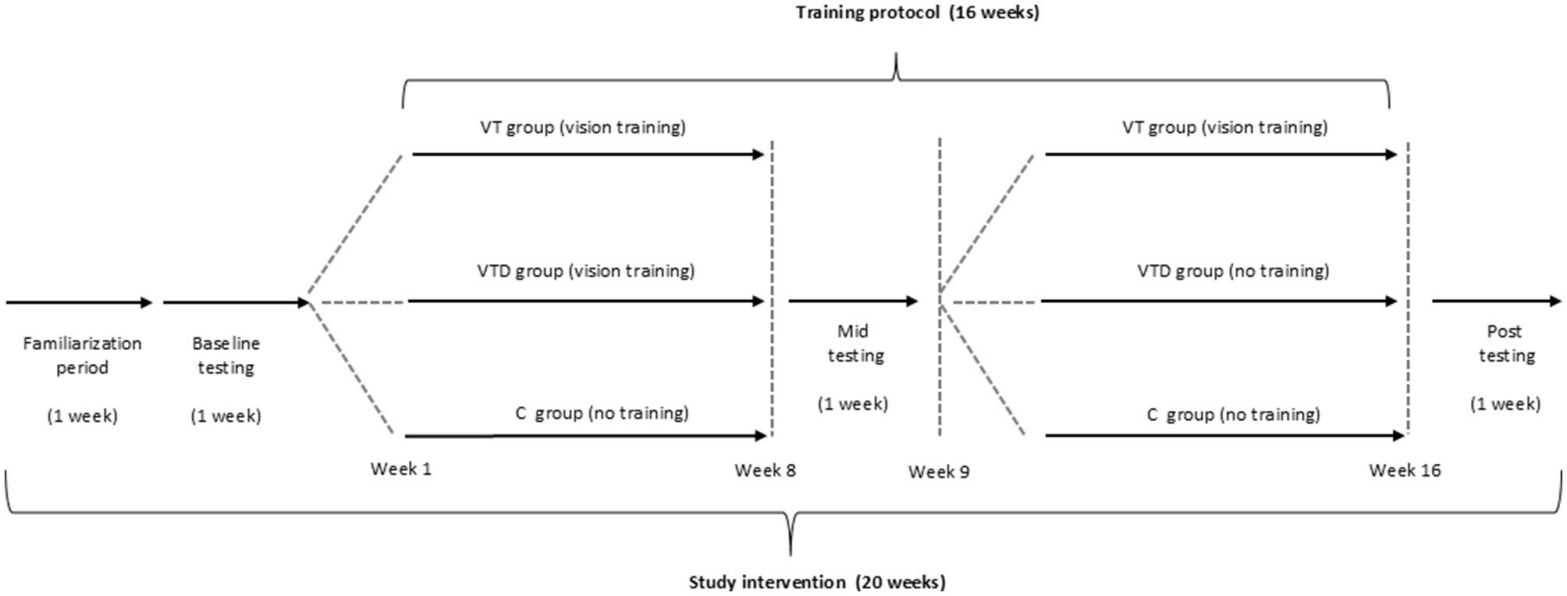
Study protocol design setting.

This study protocol is designed to investigate the effectiveness of a 16-week training program on cognitive performance and physical fitness in individuals with ID (Figure 4). The present research results are estimated to provide valuable data regarding a supervised exercise protocol to deal with IDs in a real-world context. The results will help to develop exercise prescription guidelines for individuals with ID to counteract the age-related physical and cognitive decline.

In detail, the VT group is expected to improve outcomes of both cognitive and physical domains at the end of week 16. Conversely, the VTD group during the detraining period is expected to slightly attenuate the training-induced gains achieved during the previous 8 weeks. Indeed, a short-term detraining period (i.e., 8–12 weeks) appears sufficient to rapidly lose previously gained physical adaptations (Bosquet et al., 2013), probably due to an attenuation of motor unit recruitment (Lemmer et al., 2000). When investigating the potential mediators in the association between physical fitness and cognitive function, it seems that molecular changes along with adaptations in brain structure, function and emotional factors are less impacted by short interruptions in training when compared to traditional fitness parameters (i.e., muscular strength). As a result, a detraining period may still preserve cognition and executive functions in older adults (Rodrigues et al., 2020). However, further research is necessary to clarify the effects of detraining on the potential mediators associated with physical exercise and cognitive performance in individuals with ID. Moreover, regarding the secondary aim, a positive association between cognitive outcomes and physical fitness is another expected result, given the physiological deterioration observed in the ID population (Reguera-García et al., 2023; Hartman et al., 2015; Haynes and Lockhart, 2012). Lastly, as for the tertiary aim, the expected findings are an effect of age on visual stimuli and neural changes. Thus, it is plausible to hypothesize that older individuals with ID may obtain fewer cognitive performance benefits compared to younger individuals with ID due to neurophysiological changes associated with the aging process of the visual and cognitive systems.

Interestingly, vision training combined with postural control exercises in individuals with ID holds promising practical implications across various domains. First, such a program can significantly enhance the individuals' overall functional independence by improving their ability to perform daily activities requiring cognitive and motor actions (Belva and Matson, 2013). In account to this, future perspectives could encompass a specific measurement of functional independence through the adoption of the Functional Independence Measure (FIM) scale. Potential limitations would address the lack of comparison with individuals with different disabilities (i.e., motor impairments) or the lack of an arm that would perform general physical exercise without vision training stimuli (Whitson et al., 2007).

Moreover, improved physical fitness can reduce the risk of falls by enhancing overall wellbeing (Jacob et al., 2023). Furthermore, the acquired motor skills and increased confidence can translate into greater opportunities for social interaction and meaningful relationships, fostering a sense of belonging within the individual and their community.

Overall, a vision training program has the potential to positively impact various aspects of individuals with ID in terms of spatial cognition and orientation (Henderson, 2003). From a practical perspective, educators, trainers, and therapists may benefit from incorporating visual stimuli in their exercise routines for the ID population, as this offers the possibility of directly targeting cognitive performance (Giuliani et al., 2011; Dale and Sonksen, 2002) while respecting individual differences by emphasizing body awareness and perceptions (Cavaggioni et al., 2019; Raiola et al., 2022; D'Isanto et al., 2022). Additionally, the acquisition of independence is a crucial aspect of community participation and daily life activities, and this protocol could represent an innovative solution when dealing with aging individuals with ID.

## 4 Ethics and dissemination

Informed consent forms for this study will be provided in several languages, including Italian, English, French and Dutch. All communication will emphasize the voluntary nature of participation and the fact that all necessary care will be provided. Data will be anonymised before publication. The results will be presented to interested individuals, community-dwellings who have explicitly asked for insight into the outcomes, clinicians, hospitals, private health institutions, and patient associations. Moreover, dissemination will include the study's submission to peer-reviewed journals, and results will be presented at relevant national and international congresses or symposia.
